# Comparative performance and cycle threshold values of 10 nucleic acid amplification tests for SARS-CoV-2 on clinical samples

**DOI:** 10.1371/journal.pone.0252757

**Published:** 2021-06-23

**Authors:** Miyuki Mizoguchi, Sohei Harada, Koh Okamoto, Yoshimi Higurashi, Mahoko Ikeda, Kyoji Moriya

**Affiliations:** 1 Department of Infection Control and Prevention, The University of Tokyo Hospital, Tokyo, Japan; 2 Department of Infectious Diseases, The University of Tokyo Hospital, Tokyo, Japan; Waseda University: Waseda Daigaku, JAPAN

## Abstract

**Background:**

A number of nucleic acid amplification tests (NAATs) for SARS-CoV-2 with different reagents have been approved for clinical use in Japan. These include research kits approved under emergency use authorization through simplified process to stabilize the supply of the reagents. Although these research kits have been increasingly used in clinical practice, limited data is available for the diagnostic performance in clinical settings.

**Methods:**

We compared sensitivity, specificity, and cycle threshold (Ct) values obtained by NAATs using 10 kits approved in Japan including eight kits those receiving emergency use authorization using 69 frozen-stored clinical samples including 23 positive samples with various Ct values and 46 negative samples.

**Results:**

Viral copy number of the frozen-stored samples determined with LightMix E-gene test ranged from 0.6 to 84521.1 copies/μL. While no false-positive results were obtained by any of these tests (specificity: 100% [95% CI, 88.9%-100%]), sensitivity of the nine tests ranged from 68.2% [95% CI, 45.1%-86.1%] to 95.5% [95% CI, 77.2%-99.9%] using LightMix E-gene test as the gold standard. All tests showed positive results for all samples with ≥100 copies/μL. Significant difference of Ct values even among tests amplifying the same genetic region (N1-CDC, N2) was also observed.

**Conclusion:**

Difference in the diagnostic performance was observed among NAATs approved in Japan. Regarding diagnostic kits for emerging infectious diseases, a system is needed to ensure both rapidity of reagent supply and accuracy of diagnosis. Ct values, which are sometimes regarded as a marker of infectivity, are not interchangeable when obtained by different assays.

## Introduction

SARS-CoV-2, first identified as the causative agent of a cluster of community-acquired pneumonia in Wuhan city in China, has rapidly spread worldwide in 2020. SARS-CoV-2 is highly transmissible and cause Coronavirus Disease 2019 (COVID-19) after an incubation period of around 5 days in a certain fraction of infected individuals [1]. Severity of COVID-19 ranges from mild upper respiratory infections similar to common cold to critical disease requiring intensive care. The risk of severe disease is especially high in the elderly and those with underlying diseases [1].

One of the most important means of preventing the spread of SARS-CoV-2 is early detection and isolation of infected individuals. Nucleic acid amplification tests (NAATs), mainly real-time RT-PCR assays, are considered the method with highest sensitivity for identifying infected persons. To meet the rapidly increasing demand for NAATs during the early stage of the SARS-CoV-2 pandemic, a number of NAAT kits were approved for clinical use including those receiving emergency use authorization in each country.

In Japan, a real-time RT-PCR assay to amplify two target regions (N and N2) in the nucleocapsid protein gene (N gene) of SARS-CoV-2 was developed by Japanese National Institute of Infectious Disease (NIID) and has been utilized as the reference method for the diagnosis of COVID-19 at local public laboratories [2]. The NIID N2 assay showed high sensitivity and specificity comparable to the Centers for Disease Control and Prevention (CDC) assay [3]. As of December 2020, 20 commercially-available NAAT diagnostic kits for SARS-CoV-2 have been approved and are used clinically for the diagnosis of COVID-19 in Japan. In addition, in light of shortage for supply of those approved kits, more than 30 NAAT research kits have gained emergency use authorization from Ministry of Health, Labour and Welfare for clinical use. Many of these diagnostic kits are less laborious because they do not require prior elaborate RNA extraction and incorporate a simplified RNA extraction process into the assays. Research kits have to demonstrate the ability to detect 50–200 copies/test of SARS-CoV-2 and show concordance greater than 90% with the NIID method using 10 positive and 15 negative samples at minimum for emergency use authorization. Although these research kits have been increasingly used in clinical practice, limited data is available for the diagnostic performance in real world settings.

Cycle threshold (Ct) values obtained by real-time RT-PCR assays are not only used to determine the presence of viruses in clinical specimens using pre-defined threshold value for each assay, but are also often regarded as a surrogate marker that correlates with viral load. However, it remains uncertain to what extent Ct values obtained by various real-time RT-PCR assays are interchangeable. Theoretically, they could be different depending on the amount of sample used for the reaction, RNA extraction method, target gene, and NAAT conditions and reagents.

In this study, we compared the sensitivity, specificity, and Ct values obtained by 10 different NAATs, including the ones with emergency use authorization, available in Japan.

## Materials and methods

### SARS-CoV-2 NAAT at the hospital

LightMix^®^ Modular SARS-CoV (COVID19) E-gene (Envelope gene) kit and RdRP-gene (RNA-dependent RNA polymerase gene) kit (TIB MOLBIOL, Germany) were used for real-time RT-PCR at the University of Tokyo Hospital (UTH) for diagnosis of COVID-19. Nasopharyngeal swabs were suspended in 3 mL of saline contained in a sterile tube immediately after collection and were transported to the microbiology laboratory, where the supernatant was collected after centrifugation at 15,000 rpm for 2 min. Sputum sample was suspended with equal to triple volume of phosphate-buffered saline and the supernatant was collected after centrifugation at 20,000 rpm for 30 minutes. RNA extraction and purification from the supernatant were performed with magLEAD 6gC and MagDEA Dx SV (Precision System Science Co Ltd, Japan) and 100 μL of RNA extract was obtained from 400 μL of supernatant. The remaining supernatant was frozen-stored at −80°C. Real-time RT-PCR was performed with cobas z480 system (Roche Diagnostics, Switzerland) using 10 μL of RNA extract according to the package insert. The result was reported as positive when the Ct value of the reaction for E gene or RdRP gene was 40 or less.

### Calibration curve of LightMix E-gene test

To draw the calibration curve of the LightMix E-gene test, real-time RT-PCR was performed using the positive control provided with known copy number supplied in the kit. The positive control was diluted with RNase/DNase-free 10 mM Tris buffer (pH 8–8.5) to adjust the copy number to be a 3-fold dilution series from 4.1 to 1000 copies/test. All reactions were performed three times.

### Sample selection

Frozen-stored supernatants of 69 clinical samples (23 positive and 46 negative samples) submitted to UTH microbiology laboratory from May through September 2020 were used in this study. Positive samples were selected such that they represent the samples with wide range of Ct value obtained by LightMix E-gene test. Twelve samples had Ct values of >30 (range 30.75–34.60, Sample No. 1–12) and eleven samples had Ct values of <30 (range 19.29–29.05, Sample No. 13–23) (Table 1). While reactions for E-gene was positive for all 23 positive samples, those for RdRP-gene was positive only for eight positive samples (Sample No. 13, 15, 18, 19, 20, 21, 22, and 23). Twenty-one positive samples were nasopharyngeal swab samples and the remaining two samples (Sample No. 8 and 18) were sputum samples. Forty-six negative samples (42 nasopharyngeal samples and 4 sputum samples) were randomly selected from all negative samples submitted to the microbiology laboratory.

**Table 1:** Cycle threshold value obtained by each NAAT assay for frozen-stored positive samples.

Sample No.	Cycle threshold value for original sample^a^	Viral copy number (copies/μL)	NAAT assay (target region)															
			LightMix	NIID	FUJIFILM	NCV-101	NCV-301	NCV-403	SHIMADZU	NIID	FUJIFILM	NCV-102	NCV-302	NCV-403	SHIMADZU	TaKaRa	KANEKA	LAMP^b^
			(E)	(NIID-N)	(NIID-N)	(NIID-N)	(CDC-N1)	(CDC-N1)	(CDC-N1)	(NIID-N2)	(NIID-N2)	(NIID-N2)	(CDC-N2)	(CDC-N2)	(CDC-N2)	(CDC-N1, CDC-N2)	(CDC-N1, CDC-N2)	(N, RdRP)
1	34.6	3.9	33.66	36.57	-	-	-	38.41	-	36.6	-	-	-	37.11	38.3	40	38.85	+
2	34.4	14.0	31.87	-	-	34.38	-	-	34.52	35.95	-	-	-	-	33.57	-	37.32	-
3	34.34	0.6	36.30	35.89	-	-	40	36.01	33.93	34.37	31.66	-	39.3	-	33.99	35.84	35.92	+
4	33.78	2.0	34.60	-	-	-	-	-	-	-	38.38	-	-	-	-	-	-	-
5	33.53	-	-	-	-	-	-	-	-	-	-	-	-	-	-	-	-	-
6	33.18	8.6	32.55	36.26	-	-	40	36.66	34.57	34.29	30.59	34.96	-	-	-	36.19	38.65	+
7	32.73	0.7	35.97	-	-	-	40	-	-	36.62	-	37.35	-	-	-	40	-	-
8	32.46	1.4	35.08	-	-	-	-	39.49	34.48	36.04	33.52	-	38.71	-	35.71	40	40	+
9	31.23	1.2	35.25	-	-	-	36.87	37.2	32.9	38	35.78	40	38.8	36.95	33.34	37.16	36.64	+
10	30.97	18.6	31.47	35.71	-	-	-	35.16	32.91	33.61	31.81	-	-	35.37	32.81	35.88	35.45	+
11	30.94	0.7	36.07	-	-	-	-	-	36.71	36.57	36.87	40	40	-	-	-	37.88	+
12	30.75	1.0	35.53	-	-	-	-	40	33.65	36.55	-	-	-	-	33.68	37.95	40	-
13	29.05	10.4	32.29	-	-	-	37.74	36.06	29.73	33.68	32.95	-	40	36.31	29.34	36.26	38.25	+
14	28.47	742.5	26.29	32.95	-	-	30.8	29.69	27.59	28.04	29.83	31.91	31.75	29.84	26.8	30.06	38.89	+
15	27.02	50.3	30.07	35.57	-	-	36.43	34.37	31.39	32.03	30.77	37.86	37.99	34.76	31.21	34.53	35.38	+
16	26.93	987.1	25.89	32	-	-	30.28	28.84	27.27	27.2	25.64	32.31	31.69	29.11	27.21	28.32	30.52	+
17	26.75	87.7	29.29	34.04	-	36.97	37.11	34.35	30.9	31.49	29.3	35.26	40	35.05	31.71	33.59	33.8	+
18	25.47	137.3	28.66	34.06	-	37.35	34.83	32.93	29.99	30.71	28.91	36.48	36	33.27	30.25	32.69	33.91	+
19	25.09	10644.9	22.55	29.24	29.21	35.19	30.15	26.47	24.5	23.86	24.28	31.4	31.92	26.76	24	29.16	27.62	+
20	24.99	4795.3	23.67	29.53	29.97	35.82	33.56	26.93	25.47	24.22	24.85	32.14	35.24	27.12	25.18	30.19	28.55	+
21	24.04	20348.3	21.64	27.82	28.75	35.19	26.93	25	23.14	22.25	22.96	31.31	28.78	25.16	22.44	27.7	26.09	+
22	20.04	7351.0	23.07	29.36	30.84	35.17	29.12	27.55	24.86	24.78	25.56	33.12	29.61	28.02	24.36	27	28.04	+
23	19.29	84521.1	19.64	26.18	28.85	33.65	26.51	23.92	21.76	21.06	21.93	28.9	27.62	24.72	20.86	23.68	24.5	+

^a^ Cycle threshold values for original samples were obtained with LightMix E-gene test.

^b^ Results by LAMP were presented dichotomously as positive or negative because cycle threshold values were not obtained by this test.

### Tests and kits

NIID test and tests with nine commercially-available kits including LightMix E-gene test were evaluated in this study (Table 2). In the following, the name of the kit will be written in the abbreviation listed in Table 2. Among the commercially-available kits evaluated in this study, LAMP was the only kit officially approved for clinical use and the remaining eight kits were research kits exceptionally approved for clinical use at the time of purchase of the reagents (October 2020). NCV-101, -102 were later replaced by new kits released by the same manufacturer (NCV-301, -302, and later NCV-403), and are no longer recommended for use by the manufacturer. NAATs were performed according to the package inserts of the kits. Although the primers for N2 region of N-gene from NIID and those from CDC had different nucleotide sequences, both amplify the same region. TaKaRa test and KANEKA test report single Ct value for two regions, N1-CDC and N2. magLEAD 6gC and MagDEA Dx SV were used for RNA preparation for four tests requiring prior extraction of RNA. Real-time RT-PCR was performed with cobas z480 system and loop-mediated isothermal amplification (LAMP) was performed with LoopampEXIA (EIKEN CHEMICAL, Japan). The viral copy numbers were calculated by fitting the Ct values of the samples with LightMix E-gene test to the calibration curve obtained from the reaction under the same conditions using the positive control with known viral copy number supplied in the kit.

**Table 2:** NAAT kits and assays for SARS-CoV-2 used in this study.

Kit name	Method	Manufacturer	Test abbreviation	Target regions	Regulatory status in Japan	RNA extraction prior to use	Sample Volume (μL)	Reaction Volume (μL)
LightMix^®^ Modular SARS-CoV (COVID19)	Realtime RT-PCR	TIB MOLBIOL (Germany)	LightMix	E, RdRP	Research kit^a^	Necessary	10	20
-	Realtime RT-PCR	-	NIID	NIID-N, NIID-N2	-	Necessary	5	25
SARS-CoV-2 RT-qPCR Detection Kit	Realtime RT-PCR	FUJIFILM Wako Pure Chemical (Japan)	FUJIFILM	NIID-N, NIID-N2	Research kit^a^	Necessary	5	20
SARS-CoV-2 Detection Kit NCV-101, NCV-102	Realtime RT-PCR	TOYOBO (Japan)	NCV-101, NCV-102	NIID-N, NIID-N2	Research kit^a^	Unnecessary	3	46
2019 Novel Coronavirus Detection Kit	Realtime RT-PCR	SHIMADZU (Japan)	SHIMADZU	CDC-N1, CDC-N2	Research kit^a^	Unnecessary	5	25
SARS-CoV-2 Detection Kit NCV-301, NCV-302	Realtime RT-PCR	TOYOBO (Japan)	NCV-301, NCV-302	CDC-N1, CDC-N2	Research kit^a^	Unnecessary	6	49
SARS-CoV-2 Detection Kit -Multi- NCV-403	Realtime RT-PCR	TOYOBO (Japan)	NCV-403	CDC-N1, CDC-N2	Research kit^a^	Unnecessary	8	51
SARS-CoV-2 Direct Detection RT-qPCR Kit	Realtime RT-PCR	TaKaRa (Japan)	TaKaRa	CDC-N1, CDC-N2	Research kit^a^	Unnecessary	8	50
KANEKA Direct RT-qPCR Kit “SARS-CoV-2”	Realtime RT-PCR	KANEKA (Japan)	KANEKA	CDC-N1, CDC-N2	Research kit^a^	Unnecessary	4	25
Loopamp^®^ SARS-CoV-2 Detection Kit	Loop-mediated isothermal amplification	EIKEN CHEMICAL (Japan)	LAMP	N, RdRP	Clinical diagnostics	Necessary	10	25

^a^ These kits have gained emergency use authorization for clinical use.

### Comparison of the results of NAAT for clinical samples

The sensitivity and specificity of each test were calculated using the LightMix E-gene test as the gold standard. Ct value of 40 or less was interpreted as an amplification of the target site, and a test was determined to be positive when amplification of any target site in a single test was observed. For each target site, the Ct values were statistically compared for samples with results in all kits (N-NIID (n = 5), N1-CDC (n = 14), N2 (n = 11), N1-CDC/N2 (n = 18)).

### Statistical analysis

All statistical analyses were performed with EZR (ver. 1.37) [4]. Wilcoxon paired rank sum test and Friedman test with Bonferroni adjustment were used for pairwise comparison of Ct values of two tests and more than three tests, respectively. A p-value of 0.05 or less was interpreted as statistically significant.

### Ethics

This study was approved by the local ethics committee of the University of Tokyo Hospital (3538-(11)). Patient consent was waived by the ethics committee because no patient information was used in this study and the sample was anonymized.

## Results

### Calibration curve and PCR efficiency of LightMix E-gene test

**Fig 1:**
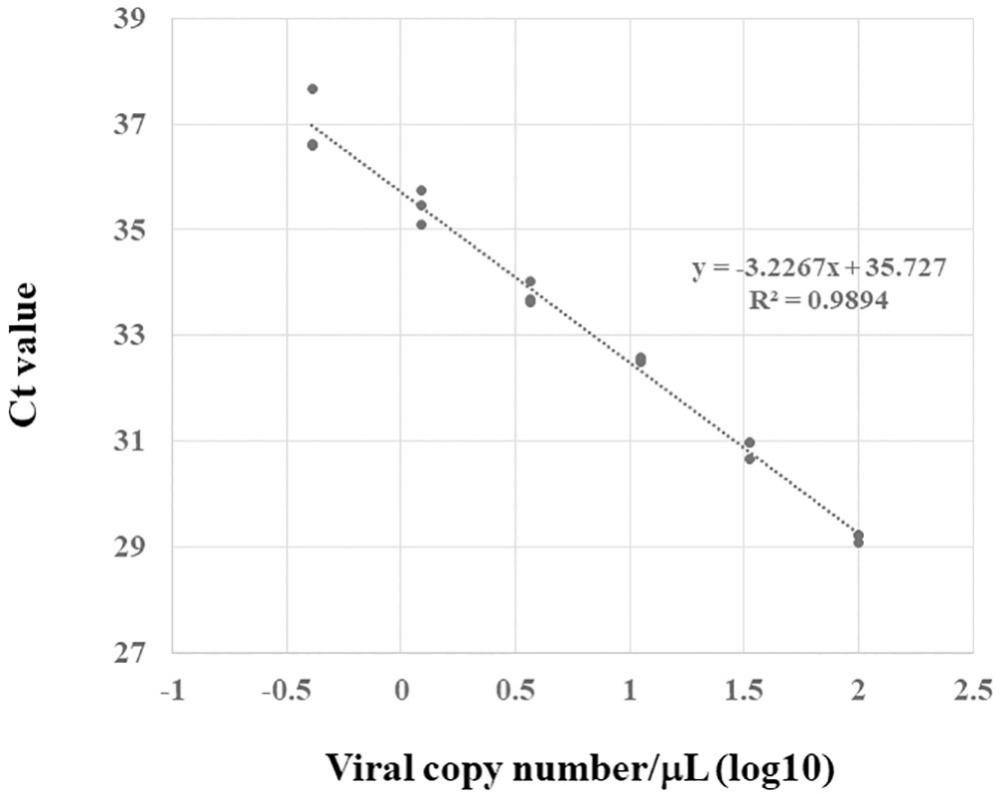
Calibration curve of LightMix E-gene test.

Calibration curve of LightMix E-gene test is presented in Fig 1. Coefficient of determination and PCR efficiency was 0.9894 and 104%, respectively.

### Performance of the detection assays for clinical samples

Sensitivity and specificity of each test (total and by target region) are presented in Table 3. One of the frozen-stored positive samples (No. 5) showed negative results in all tests, which may be due to RNA degradation in the process of cryopreservation and thawing (Table 1). Viral copy number of the frozen-stored samples determined with LightMix E-gene test ranged from 0.6 to 84521.1 copies/μL. Fourteen samples and eight samples had copy number of <100 copies/μL and ≥100 copies/μL, respectively. Although the results for the frozen-stored negative samples were negative by all tests, sensitivity of the tests ranged from 68.2% (95% CI, 45.1%-86.1%) by NCV-101, NCV-102 to 95.5% (95% CI, 77.2%-99.9%) by NIID. All tests showed positive results for all samples with ≥100 copies/μL.

**Table 3:** Diagnostic performance of NAAT assays.

Test (target region)	Positive result for samples with <100 copies/μL	Positive result for samples with ≥100 copies/μL	Sensitivity [95% CI]	Specificity [95% CI]
	(n = 69)	(n = 69)	(n = 14)	(n = 8)
NIID	13 (92.9%)	8 (100%)	95.5% [77.2%-99.9%]	100% [88.9%-100%]
NIID (NIID-N)	6 (42.9%)	8 (100%)	63.6% [40.7%-82.8%]	100% [88.9%-100%]
NIID (NIID-N2)	13 (92.9%)	8 (100%)	95.5% [77.2%-99.9%]	100% [88.9%-100%]
FUJIFILM	10 (71.4%)	8 (100%)	81.8% [59.7%-94.8%]	100% [88.9%-100%]
FUJIFILM (NIID-N)	0 (0%)	5 (62.5%)	22.7% [7.8%-45.4%]	100% [88.9%-100%]
FUJIFILM (NIID-N2)	10 (71.4%)	8 (100%)	81.8% [59.7%-94.8%]	100% [88.9%-100%]
NCV-101, -102	7 (50%)	8 (100%)	68.2% [45.1%-86.1%]	100% [88.9%-100%]
NCV-101 (NIID-N)	2 (14.3%)	6 (75%)	36.4% [17.2%-59.3%]	100% [88.9%-100%]
NCV-102 (NIID-N2)	6 (42.9%)	8 (100%)	63.6% [40.7%-82.8%]	100% [88.9%-100%]
SHIMADZU	12 (85.7%)	8 (100%)	90.9% [70.8%-98.9%]	100% [88.9%-100%]
SHIMADZU (CDC-N1)	11 (78.6%)	8 (100%)	86.4% [65.1%-97.1%]	100% [88.9%-100%]
SHIMADZU (CDC-N2)	10 (71.4%)	8 (100%)	81.8% [59.7%-94.8%]	100% [88.9%-100%]
NCV-301, -302	9 (64.3%)	8 (100%)	77.3% [54.6%-92.2%]	100% [88.9%-100%]
NCV-301 (CDC-N1)	7 (50%)	8 (100%)	68.2% [45.1%-86.1%]	100% [88.9%-100%]
NCV-302 (CDC-N2)	7 (50%)	8 (100%)	68.2% [45.1%-86.1%]	100% [88.9%-100%]
NCV-403	10 (71.4%)	8 (100%)	81.8% [59.7%-94.8%]	100% [88.9%-100%]
NCV-403 (CDC-N1)	10 (71.4%)	8 (100%)	81.8% [59.7%-94.8%]	100% [88.9%-100%]
NCV-403 (CDC-N2)	6 (42.9%)	8 (100%)	63.6% [40.7%-82.8%]	100% [88.9%-100%]
TaKaRa	11 (78.6%)	8 (100%)	86.4% [65.1%-97.1%]	100% [88.9%-100%]
(CDC-N1, CDC-N2)				
KANEKA	12 (85.7%)	8 (100%)	90.9% [70.8%-98.9%]	100% [88.9%-100%]
(CDC-N1, CDC-N2)				
LAMP (N, RdRP)	10 (71.4%)	8 (100%)	81.8% [59.7%-94.8%]	100% [88.9%-100%]

Data are No. (%) of patients unless otherwise indicated.

CI, confidence interval.

### Cycle threshold values obtained by different tests

**Fig 2:**
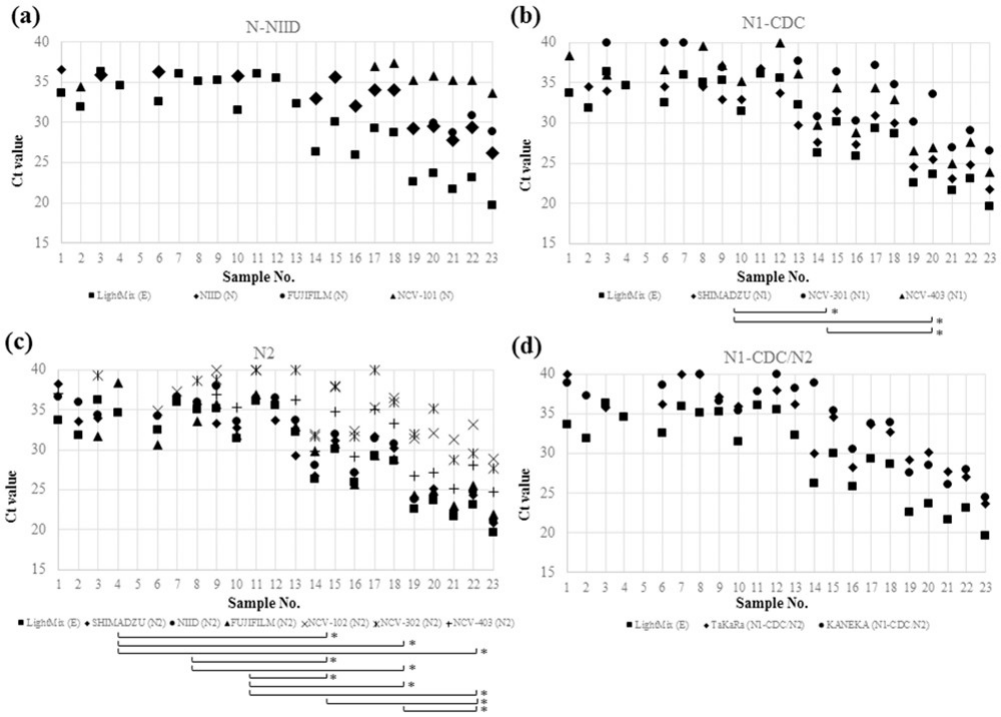
Cycle threshold values obtained by NAATs amplifying (a) N-NIID, (b) N1-CDC, (c) N2, and (d) N1-CDC/N2. The combinations of tests that showed statistically significant differences in cycle threshold values are indicated by brackets with asterisks at the right end. Cycle threshold values obtained by LightMix E-gene test were plotted in all graphs as references.

Ct values obtained by each test are shown in Table 1. As shown in Fig 2, differences in Ct values were observed even when testing for the same target in the same sample. For N1-CDC target site, all pairs of Ct values obtained by three tests showed statistically significant difference (p < 0.001 for NCV-301/NCV-403, NCV-301/SHIMADZU, and NCV-403/SHIMADZU). For N2 target site, 10 of 15 pairs of Ct values obtained by six tests showed statistically significant difference (p = 0.015 for FUJIFILM/NCV-102, FUJIFILM/NCV-302, FUJIFILM/NCV-403, NCV-102/NCV-403, NCV-102/NIID, NCV-102/SHIMADZU, NCV-302/NCV-403, NCV-302/NIID, NCV-302/SHIMADZU, NCV-403/SHIMADZU). For N-NIID target site, Ct values did not show statistically significant difference (p = 0.19 for NCV-101/FUJIFILM and NCV-101/NIID, p = 0.38 for FUJIFILM/NIID). For two tests (TaKaRa and KANAKA) that report single Ct value for two target sites, N1-CDC and N2, Ct values did not show statistically significant difference (p = 0.185).

## Discussion

In this study, we compared the diagnostic performance and Ct values of NAATs for SARS-CoV-2 approved for use in clinical diagnosis in Japan using clinical samples. Eight research kits, which have gained emergency use authorization for clinical use, showed significant difference in sensitivity and Ct values.

Overall, all tests successfully identified the presence of SARS-CoV-2 from all samples with ≥100 copies/μL. Although difference in sensitivity between tests with different target sites was noted, it was largely due to the difference in the ability for detection of SARS-CoV-2 with low copy numbers. This difference might be of little importance from the standpoint of infection control given that patients whose clinical samples have low viral load tend to have low infectivity. Nonetheless, presymptomatic and asymptomatic patients have been shown to be involved in transmission although viral load of a part of these patients may be low [5–7]. In addition, a recent study showed that individuals infected after vaccination shed less viruses [8]. Therefore, NAATs with high sensitivity very likely continue to have an important role in detecting those patients with low viral load.

Notably, LAMP test showed sensitivity comparable to real-time RT-PCR assays as previously reported, and all samples with ≥100 copies/μL were positive with this test [9]. LAMP test is a useful option for the clinical diagnosis of COVID-19 in Japan, where the devices for this test have been employed at a number of healthcare facilities for the detection of other pathogens [10].

In this study, difference in Ct values was observed even among tests amplifying the same genetic region. This is in line with the results of previous studies which analyzed Ct values obtained by different NAAT assays using the same specimens [11, 12]. Although the results of NAAT for SARS-CoV-2 is generally reported dichotomously as positive or negative in clinical settings, some advocate for semi-quantitative reporting according to Ct values (e.g., high, medium, and low) [13]. This may be reasonable considering that low Ct values correlate with positive viral culture, a marker of infectivity [14–16]. Nevertheless, these studies employing different NAAT assays presented different Ct value thresholds (e.g., 24, 32, or 35) for predicting culture positivity. Standardization of the reporting of SARS-CoV-2 NAAT results which provide information useful for clinical decision is warranted.

There are several limitations in this study. First, we included the small number of positive samples. However, we attempted to overcome this weakness by selecting samples with the wide range of viral load. Second, we performed this evaluation using preserved samples which may have reduced number of viral copies. Yet, all the samples were promptly frozen at −80°C and kept for up to 6 months until the evaluation was performed. All tests were implemented using samples with the same conditions of one freeze and one thaw. Third, LightMix E-gene test was used as a gold standard for the evaluation of other tests. This is because this test was used clinically for the diagnosis of COVID-19 at UTH during the period of sample collection and the samples used in this study were selected based on the results. LightMix E-gene test has been available from the early phase of the pandemic of SARS-CoV-2 and has been used worldwide [17, 18]. The agreement of the results by LightMix E-gene test with those by NIID-N2 and CDC test was high in previous reports [3, 19]. Lower Ct values obtained by LightMix E-gene test than those obtained by other tests and low sensitivity of LightMix RdRP-gene test in our study were in agreement with previous studies [3, 17].

In conclusion, differences in sensitivity and Ct values were found among NAAT tests approved for the diagnosis of SARS-CoV-2 in Japan. In the context of a global pandemic of emerging infectious diseases, it is important to supply diagnostic kits as soon as possible, but to balance this with quality assurance is difficult. Based on the experience of pandemic of SARS-CoV-2, the extraordinary approval process for diagnostic kits needs to be prepared in each country for the next emerging infectious disease.
