# Correction: Comparative performance and cycle threshold values of 10 nucleic acid amplification tests for SARS-CoV-2 on clinical samples

**DOI:** 10.1371/journal.pone.0314897

**Published:** 2024-11-27

**Authors:** The PLOS One Staff

There is an error in Table 3. The number of samples (n = X) are incorrect. Please see the correct Table 3 here.

**Table 3:** Diagnostic performance of NAAT assays.

Test (target region)	Positive result for samples with <100 copies/mL (n = 14)	Positive result for samples with ≥100 copies/mL (n = 8)	Sensitivity [95% CI] (n = 69)	Specificity [95% CI] (n = 69)
NIID	13 (92.9%)	8 (100%)	95.5% [77.2%-99.9%]	100% [88.9%-100%]
NIID (NIID-N)	6 (42.9%)	8 (100%)	63.6% [40.7%-82.8%]	100% [88.9%-100%]
NIID (NIID-N2)	13 (92.9%)	8 (100%)	95.5% [77.2%-99.9%]	100% [88.9%-100%]
FUJIFILM	10 (71.4%)	8 (100%)	81.8% [59.7%-94.8%]	100% [88.9%-100%]
FUJIFILM (NIID-N)	0 (0%)	5 (62.5%)	22.7% [7.8%-45.4%]	100% [88.9%-100%]
FUJIFILM (NIID-N2)	10 (71.4%)	8 (100%)	81.8% [59.7%-94.8%]	100% [88.9%-100%]
NCV-101, -102	7 (50%)	8 (100%)	68.2% [45.1%-86.1%]	100% [88.9%-100%]
NCV-101 (NIID-N)	2 (14.3%)	6 (75%)	36.4% [17.2%-59.3%]	100% [88.9%-100%]
NCV-102 (NIID-N2)	6 (42.9%)	8 (100%)	63.6% [40.7%-82.8%]	100% [88.9%-100%]
SHIMADZU	12 (85.7%)	8 (100%)	90.9% [70.8%-98.9%]	100% [88.9%-100%]
SHIMADZU (CDC-N1)	11 (78.6%)	8 (100%)	86.4% [65.1%-97.1%]	100% [88.9%-100%]
SHIMADZU (CDC-N2)	10 (71.4%)	8 (100%)	81.8% [59.7%-94.8%]	100% [88.9%-100%]
NCV-301, -302	9 (64.3%)	8 (100%)	77.3% [54.6%-92.2%]	100% [88.9%-100%]
NCV-301 (CDC-N1)	7 (50%)	8 (100%)	68.2% [45.1%-86.1%]	100% [88.9%-100%]
NCV-302 (CDC-N2)	7 (50%)	8 (100%)	68.2% [45.1%-86.1%]	100% [88.9%-100%]
NCV-403	10 (71.4%)	8 (100%)	81.8% [59.7%-94.8%]	100% [88.9%-100%]
NCV-403 (CDC-N1)	10 (71.4%)	8 (100%)	81.8% [59.7%-94.8%]	100% [88.9%-100%]
NCV-403 (CDC-N2)	6 (42.9%)	8 (100%)	63.6% [40.7%-82.8%]	100% [88.9%-100%]
TaKaRa (CDC-N1, CDC-N2)	11 (78.6%)	8 (100%)	86.4% [65.1%-97.1%]	100% [88.9%-100%]
KANEKA (CDC-N1, CDC-N2)	12 (85.7%)	8 (100%)	90.9% [70.8%-98.9%]	100% [88.9%-100%]
LAMP (N, RdRP)	10 (71.4%)	8 (100%)	81.8% [59.7%-94.8%]	100% [88.9%-100%]

The publisher apologizes for the error.
